# Transforming Clinical Research: The Power of High-Throughput Omics Integration

**DOI:** 10.3390/proteomes12030025

**Published:** 2024-09-06

**Authors:** Rui Vitorino

**Affiliations:** 1 iBiMED, Department of Medical Sciences, University of Aveiro, 3810-193 Aveiro, Portugal; rvitorino@ua.pt; 2 Department of Surgery and Physiology, Cardiovascular R&D Centre—UnIC@RISE, Faculty of Medicine, University of Porto, 4200-319 Porto, Portugal

**Keywords:** high-throughput omics, data integration, next-generation sequencing, bioinformatics, personalized medicine, biomarkers

## Abstract

High-throughput omics technologies have dramatically changed biological research, providing unprecedented insights into the complexity of living systems. This review presents a comprehensive examination of the current landscape of high-throughput omics pipelines, covering key technologies, data integration techniques and their diverse applications. It looks at advances in next-generation sequencing, mass spectrometry and microarray platforms and highlights their contribution to data volume and precision. In addition, this review looks at the critical role of bioinformatics tools and statistical methods in managing the large datasets generated by these technologies. By integrating multi-omics data, researchers can gain a holistic understanding of biological systems, leading to the identification of new biomarkers and therapeutic targets, particularly in complex diseases such as cancer. The review also looks at the integration of omics data into electronic health records (EHRs) and the potential for cloud computing and big data analytics to improve data storage, analysis and sharing. Despite significant advances, there are still challenges such as data complexity, technical limitations and ethical issues. Future directions include the development of more sophisticated computational tools and the application of advanced machine learning techniques, which are critical for addressing the complexity and heterogeneity of omics datasets. This review aims to serve as a valuable resource for researchers and practitioners, highlighting the transformative potential of high-throughput omics technologies in advancing personalized medicine and improving clinical outcomes.

## 1. Introduction

**Figure 1:**
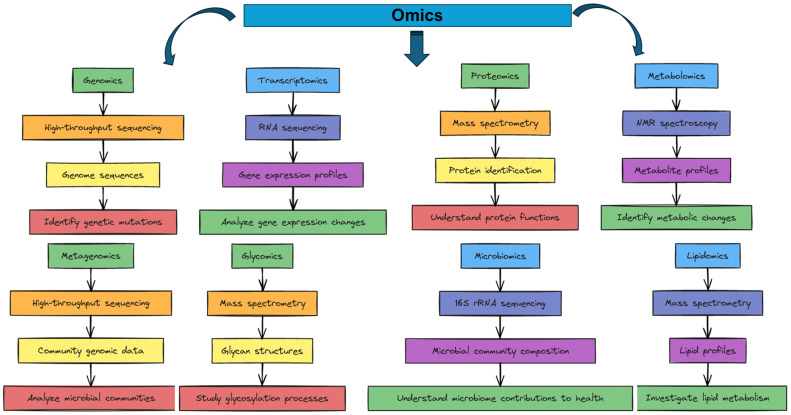
Overview of Omics Technologies.

The omics high-throughput pipeline is transforming biological research by enabling comprehensive, large-scale analysis of diverse biomolecular data. These advanced technologies generate extensive data at multiple omics levels, including genomics, transcriptomics, proteomics and metabolomics. To handle the complexity and volume of this data, sophisticated bioinformatics pipelines are required that integrate various software tools and databases to pre-process, analyze and interpret the data, forming intricate workflows (Figure 1). The integration of high-throughput omics is primarily based on two fundamental approaches: similarity-based methods and difference-based methods. Similarity-based methods aim to identify common patterns, correlations and common paths in different omics datasets. These methods are crucial for understanding overarching biological processes and identifying universal biomarkers. For example, correlation analysis evaluates the correlation between different omics levels (e.g., genomics, transcriptomics and proteomics) to find common trends and relationships and identify co-expressed genes or proteins in different datasets. Clustering algorithms, such as hierarchical clustering and k-means clustering, group similar data points from different omics datasets and uncover modules or networks of genes and proteins that work together. Network-based approaches, such as Similarity Network Fusion (SNF), construct similarity networks for each omics type and then integrate them into a single network, merging information from all omics levels to highlight commonalities and identify important biological pathways. On the other hand, difference-based methods focus on detecting unique features and variations between different omics levels, which is essential for understanding disease-specific mechanisms and for personalized medicine. Differential expression analysis compares the expression levels of genes or proteins between different states (e.g., healthy vs. diseased) to identify significant changes and recognize unique molecular signatures associated with specific conditions [1]. Variance decomposition decomposes the total variance observed in the data into components attributable to different omics levels. This helps us to understand the contribution of each omics type to the overall variability and to identify omics-specific variation. Feature selection methods, such as LASSO (Least Absolute Shrinkage and Selection Operator) and Random Forests, select the most relevant features from each omics dataset and integrates these features into a comprehensive model that captures the unique aspects of each layer [2]. Popular integration algorithms include Multi-Omics Factor Analysis (MOFA) and Canonical Correlation Analysis (CCA). MOFA is an unsupervised approach that uses Bayesian factor analysis to identify latent factors responsible for variation in multiple omics datasets and integrates the data to identify underlying biological signals. CCA identifies linear relationships between two or more omics datasets, facilitating the discovery of correlated traits and common pathways [3].

In genomic analysis (Table 1), tools such as Ensembl (https://www.ensembl.org/, accessed on 1 June 2024) and Galaxy [4] are essential. Ensembl provides comprehensive genomic data, while Galaxy [4] offers a user-friendly platform for bioinformatics workflows, including genome assembly, variant calling, transcriptomics and epigenomic analysis. For multi-omics data integration and visualization, OmicsNet and NetworkAnalyst are invaluable. OmicsNet [5] supports the integration of genomics, transcriptomics, proteomics and metabolomics data to create comprehensive biological networks. NetworkAnalyst [6] provides data filtering, normalization, statistical analysis and network visualization capabilities. These tools enable researchers to uncover new pathways and molecular mechanisms, driving advancements in precision medicine and other fields.

The integration of high-throughput omics combines data from different omics technologies to gain a comprehensive understanding of biological systems (Figure 1, Table 1). This integration is essential to unravel the complexity of cellular processes and disease mechanisms. This review explores advanced technologies and computational methods that facilitate omics integration, covering platforms such as next-generation sequencing (NGS) for genomics, RNA sequencing (RNA-Seq) for transcriptomics, mass spectrometry for proteomics and nuclear magnetic resonance (NMR) spectroscopy for metabolomics. It addresses challenges such as heterogeneity, scale and standardization in data and proposes solutions such as advanced bioinformatics tools and machine learning techniques. Key applications include automated text mining techniques such as natural language processing (NLP) to extract meaningful information from scientific literature and genomic analyses to identify biomarkers for diseases to improve diagnostic tools and personalized medicine. Integrating data from resources such as the GWAS catalog helps identify genetic variants associated with different traits, supporting biomarker discovery and therapeutic targets. Proteomics, facilitated by mass spectrometry, provides insights into protein functions and interactions, and the integration of proteomics data with other omics datasets improves our understanding of disease mechanisms. Effective integration strategies, such as early, mixed, middle, late and hierarchical integration, are essential for comprehensive insights into complex biological systems.

## 2. Comprehensive Frameworks for High-Throughput Pipeline Omics Integration

High-throughput omics integration pipelines focus on the development of comprehensive frameworks for efficiently processing, analyzing and interpreting large amounts of biological data generated by genomics, transcriptomics, proteomics and metabolomics technologies [7]. These integration pipelines address the challenges posed by the heterogeneity and complexity of multi-omics data, ensuring effective combination and meaningful insights. Advanced computational methods and bioinformatics tools are essential for this integration. Platforms such as OmicsNet and NetworkAnalyst [6] are critical for managing and analyzing multi-omics data. OmicsNet facilitates the visual analysis of biological networks by integrating genomics, transcriptomics, proteomics and metabolomics data and provides an intuitive user interface and extensive visualization options. NetworkAnalyst [6] provides robust tools for network-based visual analysis that support transcriptomics, proteomics and metabolomics data and include features for data filtering, normalization, statistical analysis and network visualization, all accessible without programming knowledge.

These integrated pipelines streamline workflows from data acquisition to analysis, promoting the discovery of complex biological relationships and pathways. By building detailed molecular networks, researchers gain deeper insights into cellular functions and disease mechanisms, facilitating the identification of novel biomarkers and therapeutic targets and ultimately advancing precision medicine [8]. In addition, integrated multi-omics pipelines translate high-dimensional biological data into actionable knowledge by enabling the simultaneous investigation of different molecular layers and providing a holistic view of biological systems. This integrative approach is particularly valuable in the study of diseases such as cancer, where understanding the interplay between genetic mutations, changes in gene expression, protein modifications and metabolic shifts is critical to the development of effective treatments. These advanced pipelines also improve the reproducibility and accessibility of next-generation sequencing analyses, benefiting a wide range of research applications (Table 2) [9,10].

### 2.1. Key Components and Technologies

High-throughput omics technologies have revolutionized biological research by enabling comprehensive analysis of the molecular components in cells. These technologies rely on several advanced methods and key components, each of which critical to capturing the complexity of biological systems. At the center of these technologies are data generation platforms, such as next-generation sequencing (NGS) for genomics, RNA sequencing (RNA-Seq) for transcriptomics, mass spectrometry for proteomics and nuclear magnetic resonance (NMR) spectroscopy for metabolomics. These platforms enable the rapid and large-scale collection of data on different molecular entities, providing a detailed view of the biological landscape [9,10].

NGS has revolutionized genomics by enabling the rapid sequencing of entire genomes at high speed and low cost. This technology is critical for identifying genetic variation, understanding the contribution of genes to disease and exploring evolutionary relationships. RNA-Seq provides comprehensive gene expression data by sequencing the RNA present in a sample and is therefore essential for transcriptomic studies. It enables researchers to quantify the level of gene expression, identify splice variants and investigate gene regulatory networks. In proteomics, mass spectrometry is the cornerstone technology for identifying and quantifying proteins in complex mixtures. It reveals changes in protein expression, post-translational modifications and protein–protein interactions, providing insights into cellular functions and signaling pathways. NMR spectroscopy, which is essential in metabolomics, provides detailed insights into the mechanisms of small molecule metabolites present in biological samples. It enables the investigation of metabolic pathways, interactions and changes associated with various physiological and pathological conditions [11].

In addition to data generation, high-throughput omics requires a robust computational infrastructure for data analysis and integration. The extensive and heterogeneous nature of omics data requires advanced computational resources and bioinformatics frameworks capable of processing, integrating and visualizing this data. Platforms such as G-language Genome Analysis Environment [12] and anvi’o [13] provide comprehensive tools for gene prediction, pathway mapping and interactive data visualization. These platforms are essential for managing large-scale omics data and enable researchers to extract meaningful insights from complex datasets. The development of new computational techniques further enhances the ability to integrate and interpret complex omics datasets. Techniques such as blockwise sparse principal component analysis and network-based methods overcome challenges such as variable redundancy and computational instability. These methods ensure accurate and reliable data integration, enabling researchers to construct detailed molecular networks and gain new biological insights [14,15]. For example, the use of advanced clustering algorithms and network-based approaches, such as Similarity Network Fusion (SNF), enables the integration of multi-omics data into a unified framework that highlights common pathways and interactions across different molecular layers [16,17].

Effective data integration strategies are critical to advancing our understanding of complex biological systems. Integrative tools such as OmicsNet [18] and NetworkAnalyst [6] play a central role in managing and analyzing multi-omics data. OmicsNet facilitates the visual analysis of biological networks and supports the integration of genomics, transcriptomics, proteomics and metabolomics data to create comprehensive biological networks. Its intuitive user interface and extensive visualization options make it accessible to researchers, enabling them to explore complex interactions within biological systems. NetworkAnalyst, on the other hand, offers powerful features for network-based visual analysis of multi-omics data. This platform supports the integration of data from different omics layers and provides robust tools for data filtering, normalization, statistical analysis and network visualization. Thanks to its user-friendly design and extensive online tutorials, NetworkAnalyst is accessible to researchers with varying levels of computer literacy.

These integrative tools not only streamline the workflow from data acquisition to analysis, but also greatly enhance the discovery of complex biological relationships and pathways. By constructing detailed molecular networks, researchers can gain a deeper understanding of cell function and disease mechanisms. This comprehensive approach facilitates the identification of novel biomarkers and therapeutic targets, ultimately advancing precision medicine and other fields.

**Table 2:** Concept And Need of High-Throughput Omics.

Aspect	Description	Examples	References
Concept	High-throughput omics technologies encompass genomics, transcriptomics, proteomics and metabolomics, enabling comprehensive analysis of molecular components.	Genomics: DNA sequencing, Transcriptomics: RNA sequencing, Proteomics: Mass spectrometry, Metabolomics: NMR spectroscopy.	[19]
Need	The complexity and heterogeneity of biological systems necessitate advanced methods to capture molecular interactions and dynamics.	Multifactorial diseases like cancer require comprehensive data to understand gene–protein–metabolite interactions.	[20]
Benefits	Provides detailed views of biological systems, identifies novel biomarkers and facilitates personalized medicine.	Improves disease understanding, targeted therapeutic strategies and customized treatment plans.	[21]
Challenges	Managing and integrating vast, heterogeneous datasets and developing accurate computational models.	Data heterogeneity, computational resource requirements and the need for advanced bioinformatics tools.	[21]

### 2.2. Challenges and Opportunities

High-throughput omics technologies offer unprecedented opportunities to advance biological research, but they also pose significant challenges. One of the biggest challenges is integrating and interpreting the vast and heterogeneous datasets that these technologies generate. The complexity and scale of omics data require sophisticated computational methods and bioinformatics infrastructures to effectively process and understand the information. Issues such as data heterogeneity, noise and the lack of standardized data formats further complicate the integration process. Advanced computational solutions such as Omics Integrator software and network-based approaches have been developed to overcome these challenges by integrating different datasets and uncovering molecular pathways. However, a critical bottleneck for many researchers remains the need for improved training in data analysis and bioinformatics. Despite these challenges, high-throughput omics technologies offer numerous opportunities for transformative advances in biological research and personalized medicine [22]. The integration of multi-omics data provides a more comprehensive understanding of biological systems and enables the identification of new biomarkers and therapeutic targets. This integrative approach is particularly valuable in the study of complex diseases such as cancer, where understanding the interactions between different molecular components is crucial. In addition, the development of cloud computing and big data analytics offers promising solutions for storing, analyzing and sharing omics data on a large scale [23]. These technological advances facilitate the creation of detailed molecular atlases and the development of predictive models that can improve clinical outcomes and advance the field of precision medicine (Table 3).

Over the past decade, omics technology has undergone significant advancements, evolving from its initial focus on cataloguing genes, proteins and SNPs to performing disease-specific, in-depth analyses of various aspects of genomics, including meta-genetics, protein–protein interactions, modifications and pathway mapping. Large-scale genome-wide association studies and high-throughput techniques have become more efficient and productive in exploring previously uncharted biological systems [24]. Discovery approaches now utilize multiplex technologies, such as rapid sequencing and advanced mass spectrometry instruments, with better resolution to detect complex protein mixtures. Targeted approaches that use mass spectrometry technology to monitor multiple reactions enable rapid quantification of multiple peptides without the need for antibodies. Despite its complexity, omics technology has brought us closer to understanding the final clinical phenotype in complex diseases where multiple genes, organs and environmental influences are likely to be involved [11].

Translational research in disease greatly benefits from omics approaches, as these technologies can map entire cellular–molecular pathways to guide in vivo studies and validate bedside research. The interrelated issues of reproducibility, noise and the perception of omics as “pure fishing” are important to address. The problem of reproducibility stems from noise, and the existence of noisy datasets that cannot be reproduced, fuels this perception. Careful experiment design, targeted biological questions and appropriate interpretation and validation of results are needed to address this criticism. The core problem with omics methodology is reproducibility. A healthy skepticism about reproducibility has driven genomics research and led to the derivation of training sets from well-characterized cohorts and validation in separate cohorts. However, proteomics presents unique challenges due to its inherent diversity and instability compared to the genome. Proteomics studies are sensitive to changes in sample preparation protocols and instrumentation, which can introduce external noise into the experimental data. Well-designed and well-controlled experiments can mitigate these challenges by improving reproducibility through standardized workflows [25].

Given the dynamic nature of proteins, reproducibility in proteomics may require a new perspective. Unlike the relatively stable genome, the proteome changes over time and under different conditions. This variability can be seen as an opportunity to gain more comprehensive biological insights. For example, studying a single organism with a good phenotype over time may provide more reliable targets for generating hypotheses than pooled heterogeneous samples. In proteomics, the concept of “reproducibility” may need to be reconsidered as “continuous convergence”, where the focus is on consistent detection of biological signatures over time rather than exact replication of results. Innovative bioinformatics and mathematical tools can aid in the storing, visualization and interpretation of these dynamic datasets and ensure that meaningful conclusions can be drawn despite the biological noise [25,26]. All biological systems generate noise. Early targeted approaches focusing on a single or a few biomarkers have produced successful clinical markers. However, the challenge in the omics era is to filter out a signal from the vast amount of data generated. Better instruments and omics technologies have not always translated into more clinically relevant biomarker discoveries, possibly due to the overfitting of data and the variable quality of samples and instruments. To counteract noise, omics studies should leverage the sensitivity of latest technologies to investigate specific pathways and interactions in smaller cohorts, with each individual serving as its own control. This approach can help discover new signals and reduce confounding variables. The study of noise itself can also provide valuable insights into biological variability and homeostasis and help to redefine the criteria for reproducibility in the context of biological noise (Table 3) [27].

The integration of genome and proteome analyses is a crucial aspect of high-throughput omics that enables a comprehensive understanding of biological systems. Genome analysis provides insights into the DNA sequence and its variations, while proteome analysis provides information on the expression, modification and interaction of proteins. The integration of these two datasets can reveal how genetic variations affect protein expression and function, and thus elucidate the molecular mechanisms underlying various diseases. For example, the integration of genomic and proteomic data has been shown to improve the understanding of cancer biology, allowing researchers to identify potential biomarkers and therapeutic targets with greater accuracy. Tools such as the Omics Integrator and methods such as network-based integration help to combine these datasets and facilitate the construction of detailed molecular interaction networks [28,29].

In addition, the integration of genomic and proteomic data has significant implications for personalized medicine. By correlating genetic variants with protein expression patterns, researchers can develop more precise diagnostic tools and treatment strategies tailored to individual patients. In colorectal cancer research, for example, integrated proteogenomic analyses have identified specific protein signatures that are associated with different clinical outcomes, improving patient stratification and facilitating treatment decisions. This approach not only increases the predictive power of genomic data, but also provides a functional context that helps prioritize candidate genes and proteins for further investigation. Bioinformatics platforms, like iProClass [30] and iProXpress [31], facilitate the integration and functional annotation of datasets, enabling comprehensive analyses and meaningful biological insights [29,32]. For instance, iProClass [30] offers detailed data on protein sequences, structures, functions and pathways, facilitating the integration and analysis of complex proteomics datasets.

## 3. Case Studies and Applications

### 3.1. Automatic Text Mining in Biomedical Research

Automated text mining in biomedical research uses advanced computer techniques to extract meaningful information from the vast and rapidly growing body of scientific literature. The process uses algorithms to identify and retrieve relevant data, facilitating the synthesis of new insights from large and diverse datasets. The most important applications for text mining include the discovery of links between genes and diseases, the identification of drug targets and the annotation of gene functions. Techniques such as natural language processing (NLP), machine learning and deep learning tackle the complexity of biomedical texts. Thus, tools such as BeFree effectively extract valuable relationships between genes and diseases from the literature and advance personalized medicine and translational research [33]. BeFree uses NLP to analyze scientific texts and identify associations between genes and diseases, helping researchers uncover connections that might be missed by manual review alone. Similarly, the PubTator tool [34] provides annotations for biomedical entities such as genes, diseases and chemicals in the text, making it easier to extract relevant information from the large corpora of biomedical literature (Table 4).

An illustrative example of the application of text mining in biomedical research is myotonic dystrophy type 1 (DM1), an autosomal dominant genetic disease caused by the abnormal expansion of unstable CTG repeats in the 3’ untranslated region of the myotonic dystrophy protein kinase (DMPK) gene. DM1 primarily affects skeletal muscle, leading to myotonia, progressive distal muscle weakness and atrophy, but it also affects other tissues and systems such as the heart and central nervous system. While some studies have proposed potential therapeutic strategies for DM1, significant questions remain, particularly regarding the role of metabolic and mitochondrial dysfunction in its pathogenesis. It is therefore essential to identify the molecular targets associated with metabolic processes in DM1. To this end, a bibliometric analysis was conducted to identify relevant articles that combined DM1 with metabolic/metabolism terms. The articles were then subjected to further analysis using VOSviewer, an unbiased automatic text mining software (VOSviewer 1.6.20). This approach yielded a list of metabolic-related molecular targets for DM1, which were then compared with genes previously linked to DM1 in the DisGeNET database. This comprehensive analysis has led to the identification of new molecular targets, which will potentially enhance our understanding and treatment of DM1 [39]. Another practical example is the study of prostate cancer (PCa), the most common non-cutaneous cancer in men. The current diagnostic tools have limited accuracy and are invasive, creating a need for new, non-invasive biomarkers. Urine, a non-invasive sample containing prostate secretions, is an excellent source of potential PCa markers. VOSviewer’s automated text mining functionality was employed to retrieve and create coincidence networks of terms associated with PCa. To supplement these findings, data from DisGENET, a repository of PCa associations, and a recent bioinformatics analysis integrating all differentially expressed proteins identified in the tumor tissue and the urine of PCa patients, were also included. The results were then linked to gene expression data from the Gene Expression Omnibus (GEO) database [40] in order to establish a correlation between gene and protein content. The results of this study indicate that AXIN2, GSTM2, KLK3, LGALS3, MSMB, PRTFDC1 and SH3RF1 are significant entities associated with PCa. In particular, the KLK, LGALS3 and MSMB proteins were identified in a previous bioinformatics analysis, and a correlation was observed between gene and protein expression levels. The effectiveness of the proposed methodology was validated by the identification of elevated urinary galectin-3 levels in PCa patients relative to individuals without cancer. [41].

The aforementioned examples demonstrate the significant potential of text mining in biomedical research to facilitate the acquisition of novel insights and the identification of biomarkers, thereby advancing the understanding and treatment of complex diseases. The advantages of automated text mining in biomedical research are numerous and diverse. The automation of text data extraction and analysis through text mining markedly accelerates the pace of biomedical research, enabling researchers to maintain pace with the exponential growth of scientific publications and derive actionable knowledge from them. It facilitates a reduction in the time and effort typically required to review vast amounts of literature, thereby enhancing the ability to remain apprised of the latest research developments. Furthermore, text mining can identify patterns and relationships that may be obscured during manual reading, thereby enhancing the discovery process. Nevertheless, the utilization of automated text mining is not without its own set of challenges. A significant challenge is the inherent complexity and variability of biomedical language. The same concept can be conveyed in a multitude of ways, and the context in which a term is used can significantly alter its meaning. It is therefore essential that text mining tools are highly sophisticated in order to accurately interpret and extract relevant information. Furthermore, it is of paramount importance to guarantee the quality and precision of the extracted data, as inaccuracies in data extraction can result in erroneous conclusions.

### 3.2. Genomic Analysis and Biomarkers

Genome analysis has become an important tool in the identification and use of biomarkers for various diseases. Biomarkers are biological molecules that indicate the presence or state of a disease. Genomic biomarkers are particularly useful because they can reveal genetic predispositions and variations associated with disease states. In cancer research, for example, genomic biomarkers have helped to classify patients into different risk categories, predict responses to treatment and develop personalized therapeutic strategies. The integration of genomic data with clinical outcomes has enabled the development of accurate diagnostic tools to identify mutations and gene expression patterns associated with cancer prognosis and treatment outcomes [42,43].

Several advanced techniques and tools are required for the identification and validation of genomic biomarkers. High-throughput sequencing technologies, such as next-generation sequencing (NGS), enable comprehensive genomic profiling of tumors and other diseased tissues. This approach has facilitated the discovery of biomarkers that can predict disease susceptibility, progression and response to therapy. For example, genomic biomarkers have been successfully used to predict antibiotic resistance in pathogens, improving the efficacy of treatment strategies and reducing the spread of resistant strains [44]. Computational tools and machine learning algorithms have been developed to analyze large genomic datasets, identify significant biomarkers and interpret their biological significance. These advances not only improve diagnostic accuracy, but also open new avenues for targeted drug development and precision medicine [45]. Furthermore, the integration of genomic biomarkers into clinical practice has shown great promise in improving patient outcomes. By incorporating genomic data into standard clinical workflows, healthcare providers can offer more personalized treatment plans tailored to the individual patient’s genetic profile. This approach has been particularly successful in oncology, where genomic biomarkers can identify patients who are likely to benefit from certain targeted therapies, thereby optimizing treatment efficacy and minimizing adverse effects. The development of genomic biomarkers continues to advance, with ongoing research focused on improving their predictive power and clinical utility. As these biomarkers become more widely validated and integrated into routine clinical practice, they have the potential to change the landscape of disease diagnosis, prognosis and treatment [46,47].

The processing of genomic data involves several critical steps to transform raw sequencing data into meaningful biological insights. This process begins with the collection and storage of large amounts of data generated using high-throughput sequencing technologies. Techniques such as next-generation sequencing (NGS) generate large datasets that require efficient data management systems to handle their volume and complexity. Initial processing steps include quality control and alignment of sequencing data to a reference genome, which are crucial for accurate post-sequencing analysis. Tools like GenPipes facilitate [48] these tasks by providing a flexible, scalable framework for multi-stage workflows optimized for high-performance computing clusters and cloud environments. These systems enable the integration of various genomic applications such as RNA-Seq, ChIP-Seq and DNA-Seq and ensure that data is processed efficiently and accurately [48,49].

Advanced genomic data processing also includes sophisticated computational methods to analyze and interpret the data. Signal processing techniques such as genomic signal processing (GSP) convert DNA sequences into numerical representations and enable the application of digital signal processing methods to detect patterns and anomalies in the genomic data. This approach facilitates tasks such as gene prediction, clustering and feature extraction and provides deeper insights into genomic functions and disease mechanisms. For example, methods such as k-mer analysis and machine learning algorithms can identify significant genomic variations associated with specific phenotypes or diseases [48,49]. Furthermore, the integration of these computational techniques with statistical tools improves the accuracy and reliability of the analysis, allowing for the discovery of new biomarkers and therapeutic targets. The development of tools such as the Genomic Region Operation Kit (GROK, http://csbi.ltdk.helsinki.fi/grok/, accessed on 1 June 2024) is an example of progress in this area. It provides sophisticated interfaces for different programming languages and supports the major genomic file formats for flexible and comprehensive data processing [50,51].

In the field of proteogenomic analysis, several databases have proven to be key resources, especially for pathway integration and biomarker discovery from large datasets derived from RNAseq and proteomic analyses. The Clinical Proteomic Tumor Analysis Consortium (CPTAC) stands out as a key player in this area [48]. CPTAC offers an extensive repository of proteomic and genomic data across multiple cancer types, providing a rich resource for researchers looking to integrate multi-omics data. By utilizing CPTAC data, researchers can identify potential biomarkers and therapeutic targets by correlating protein expression profiles with genomic alterations. This comprehensive dataset enables the exploration of signaling pathways involved in cancer and facilitates the development of more targeted and effective therapies. The integration of proteomic data with genomic and transcriptomic information through CPTAC has significantly improved our understanding of cancer biology and treatment responses [48]. In addition to CPTAC and other databases, several genome browsers offer advanced data processing features that are worth mentioning. The UCSC Genome Browser (https://genome.ucsc.edu/, accessed on 1 June 2024), developed by the University of California, Santa Cruz, provides a comprehensive and customizable platform for visualizing genomic data. It supports the integration of various data types, including DNA sequences, gene annotations and epigenomic markers. Another notable tool is the Integrative Genomics Viewer (IGV) [52], which allows researchers to interactively explore large genomic datasets, including RNAseq and whole genome sequencing data. IGV’s user-friendly interface and powerful visualization options make it an invaluable resource for detailed genomic analysis. Both UCSC and IGV facilitate the complex processing and interpretation of genomic data, contributing significantly to progress in biomedical research.

One example of the integration of high-throughput genomic data is the analysis of colorectal cancer through proteogenomic characterization. Researchers integrated proteomic and genomic data from The Cancer Genome Atlas (TCGA) (https://www.cancer.gov/tcga, accessed on 1 June 2024) to identify novel proteomic subtypes of colorectal cancer. This study demonstrated that proteomic data provide additional insights beyond what genomic data alone can offer, including the identification of new molecular subtypes and potential therapeutic targets. The integration of these datasets highlighted specific proteins and phosphorylation events that were not apparent from genomic data, leading to a more comprehensive understanding of the disease and enabling more precise therapeutic interventions [53]. For example, in a proteogenomic study of a colorectal cancer cohort, a comparative proteomic and phosphoproteomic analysis of paired tumor and adjacent normal tissues was performed. This analysis provided a comprehensive catalog of colorectal cancer-associated proteins and phosphosites and identified both known and novel biomarkers, drug targets and cancer/testis antigens [54]. The integration of proteogenomics data prioritized genome-derived targets, such as copy number drivers and mutation-derived neoantigens, providing new insights. In particular, phosphoproteomics data linked Rb phosphorylation to increased proliferation and decreased apoptosis in colorectal cancer, suggesting targeting Rb phosphorylation as a therapeutic strategy. In addition, proteomics showed that decreased CD8 T cell infiltration correlated with increased glycolysis in microsatellite instability-high (MSI-H) tumors, suggesting that glycolysis is a potential target to improve the efficacy of immune checkpoint blockade in MSI-H tumors. This study demonstrates how proteogenomics can reveal new biological insights and therapeutic opportunities [54].

Another remarkable resource is the Human Protein Atlas (https://www.proteinatlas.org/, accessed on 1 June 2024), which maps protein expression in various tissues and organs. This database is invaluable for integrating protein expression data with other omics datasets, such as RNAseq, to identify tissue-specific biomarkers. Similarly, the PhosphoSitePlus database [55] provides detailed information on post-translational modifications, such as phosphorylation sites, which are critical for understanding signaling pathways and regulatory mechanisms. ProteomicsDB [56] provides a comprehensive platform for human proteomics data, enabling the integration of proteomics with genomics and metabolomics data to uncover functional insights and interactions between metabolic pathways. In addition, the PRIDE (Proteomics Identifications Database) [57] repository supports the submission and retrieval of mass spectrometry-based proteomics data and promotes data sharing and integrative analysis. Together, these databases enhance the capacity for integrative multi-omics research by driving discoveries in biomarker identification and pathway elucidation, advancing the field of precision medicine.

In another study, Ma et al. (2018) analyzed proteomes and performed whole exome and transcriptome sequencing and single nucleotide polymorphism array profiling on normal colorectal tissue, primary colorectal carcinoma (CRC) and matched metastatic liver tissue from two groups of triplet samples. They identified 112 correlating molecules associated with CNV mRNA proteins, including upregulated COL1A2 and BGN, which are associated with prognosis, and four major hot spots (chromosomes X, 7, 16 and 1) that influence global mRNA abundance in CRC liver metastases. Two sites, DMRTB1R202H and PARP4V458I, were frequently mutated only in the liver metastases cohort and showed dysregulated protein abundance. The study also found that the number of mutant peptides has prognostic potential and that somatic variants with increased protein abundance, such as MYH9 and CCT6A, have clinical significance. This proteogenomic characterization and integrative genomic analysis offers a new approach to understanding liver metastasis in colon and rectal cancer [58].

The utilization of integrated multi-omics methodologies can also elucidate the intricate mechanisms underlying complex diseases such as Alzheimer’s. The OmicPredict framework [18], which combines deep learning with feature selection methods, has been successfully applied to datasets related to Alzheimer’s disease, breast cancer and the novel coronavirus. This approach has demonstrated high prediction accuracy and effectively identified significant biomarkers from combined omics datasets, aiding in early disease prediction and patient stratification. By integrating genetic, transcriptomic and proteomic data through machine learning models, researchers have significantly enhanced the understanding of disease mechanisms and the development of personalized treatment strategies [18].

One of the most notable examples of the successful integration of omics is in cancer research. For example, studies integrating genomic, transcriptomic and proteomic data have led to significant advances in the understanding of cancer biology. By combining these datasets, researchers can identify new biomarkers and therapeutic targets and gain deeper insights into the mechanisms of cancer progression. The use of multi-omics approaches has been shown to be particularly effective in stratifying cancer subtypes and predicting patient outcomes, leading to more personalized and effective treatment strategies [59]. The integration of genetic variants, DNA methylation and gene expression data in bladder cancer research led to the identification of 48 genes significantly associated with both SNPs and CpGs. Remarkably, 75% of these results could be replicated in an independent dataset. This study highlights the opportunities that arise from combining different omics data types to uncover complex genetic mechanisms involved in disease and emphasizes the importance of integrative statistical methods for robust biological discoveries [60,61]. The integration of single-cell multi-omics data has also shown remarkable success, particularly in identifying new cell types and understanding cellular differentiation processes. By integrating data from single-cell RNA sequencing and single-cell proteomics, researchers have been able to create detailed cell atlases that elucidate cell states and functions in tissues. This approach has been instrumental in revealing the complexity of the cellular ecosystem in both healthy and diseased states, opening up new possibilities for targeted therapeutic interventions [62]. These studies illustrate the profound impact of integrating multiple omics datasets on understanding complex biological systems and improving clinical outcomes.

### 3.3. Role of the Genome-Wide Association Studies (GWAS) Catalog

The Genome-Wide Association Studies (GWAS) catalog plays a crucial role in understanding the genetic basis of complex diseases by providing a comprehensive, curated collection of genetic variants associated with different traits and diseases. Managed by the National Human Genome Research Institute (NHGRI) (https://www.loc.gov/item/lcwaN0031115/, accessed on 1 June 2024) and the European Bioinformatics Institute (EBI) [63], the catalog contains data from thousands of published GWAS [64] covering a wide range of phenotypes. This invaluable resource enables researchers to explore and identify genetic variants that contribute to disease susceptibility, aiding the discovery of new biomarkers and therapeutic targets. The GWAS catalog is continuously updated with new studies and associations, ensuring that researchers have access to the latest information in the field [64,65]. The GWAS catalog [64] improves the usability of genetic data through its advanced search capabilities and integration with other genomic resources. Tools such as the GWAS Integrator and the Phenotype-Genotype Integrator (PheGenI) [66] facilitate the exploration of genetic associations by linking GWAS results with relevant annotations and functional data from different databases. These integrative tools allow researchers to perform more comprehensive analyses that facilitate the interpretation of the biological significance of genetic variants. In addition, the catalog’s user-friendly web interface and RESTful API support high-throughput data access and analysis, allowing researchers to efficiently query and utilize the extensive dataset. This integration not only supports the identification of genetic factors underlying complex traits, but also accelerates the translation of GWAS results into clinical applications [64,67].

Genomic analysis and biomarker identification are central to disease research and have transformed our ability to diagnose, treat and understand complex diseases at the molecular level. Genomic biomarkers identified using advanced sequencing technologies and bioinformatics tools provide critical insights into genetic predispositions and variations that contribute to disease. In cancer research, for example, genomic biomarkers can categorize patients into subgroups based on their genetic profiles, helping to predict disease progression and response to treatment. This stratification is achieved through techniques such as gene expression profiling and single nucleotide polymorphism (SNP) analysis, which reveal molecular patterns associated with specific cancer types and stages. These findings facilitate the development of personalized treatment plans and targeted therapies, thus improving clinical outcomes [42,68,69]. The study of gene abnormalities and the networks of gene expression responsible for tumor development and progression is the focus of genomics, an interdisciplinary field [70]. GWAS aim to link specific genetic variations to disease [71]. For example, GWAS are used extensively to identify gene expression networks associated with tumor development and progression, to find new biomarkers for diagnosis and prognosis and to understand the molecular basis for personalized therapies (Table 5). However, GWAS have shown limited accuracy in reflecting the genetic diversity of the general population, potentially exacerbating existing inequalities in cancer. Population-specific variants may go unnoticed, and the penetrance of newly discovered genes and risk associations may not be accurately extrapolated to people from different ancestral backgrounds. This problem often occurs because the samples used in genomic studies do not represent all genetic structures. To overcome this problem, genomic researchers must be aware of the potential discrepancies that arise from studying a narrow range of genetic profiles and strive to improve the representativeness of their samples [72,73,74]. Despite these limitations, GWAS provide valuable insights into the underlying biological mechanisms and pathways associated with diseases such as cancer, aiding in the development of new treatments and therapies [74,75]. Advances in bioinformatics have enabled the development of powerful computational tools for analyzing large datasets from genomic studies. These tools integrate data from multiple sources, including transcriptome, proteome and metabolome data to provide a comprehensive picture of the underlying biological processes associated with diseases such as cancer.

Due to stringent genome-wide detection thresholds, many variants with low frequency or low to moderate impact may remain undetected in GWAS. Prioritizing variants based on functional annotations and epigenetic landmarks may increase detection performance. For example, Biswas et al. (2020) constructed a gene-level network that integrates prior knowledge and GWAS results by using gene-level annotations, such as pathways and Gene Ontology (GO) terms, to identify and characterize gene clusters considering their biological relationships [76]. They used a graph-based machine learning algorithm to prioritize candidate genes based on the gene network. Here, each gene is scored according to its connectivity in the network, which is determined by its annotations, connectivity to other genes and previous GWAS results. This method helps to identify the genes with the highest score for prioritizing variants for follow-up analyzes [76].

Typically, disease-causing variants are found near genes involved in biological pathways that are crucial to disease processes. Applying this information in a prioritized scan can increase the ability to discover loci belonging to genes grouped in a few distinct pathways. A computationally scalable system, GKnowMTes (Genomic Knowledge-guided Multiple Testing) [76], based on penalized logistic regression, was developed to enable a prioritized GWAS scan with many gene-level annotations. This method works with whole genome data at the summary level and a user-selected list of pathways and prevents overfitting by adaptively reweighting the input *p*-values with pathway enrichments learned from the data by cross-validation [76]. This approach ensures that the model does not become too specific, demonstrating its applicability using whole-genome simulations and publicly available GWAS datasets [76]. The advantage of GKnowMTest is its applicability to many pathways and genes, enabling comprehensive genome exploration, adaptive learning of weights and robustness to heterogeneity in the data, making it suitable for large-scale analyses such as on whole-genome datasets.

**Table 5:** Applications of Genomic Analysis and Biomarker Identification in Disease Research.

Disease Area	Genomic Analysis Techniques	Biomarker Identification	Advantages	Limitations	References
Cancer	Whole Genome Sequencing (WGS), Whole Exome Sequencing (WES), Targeted Sequencing	Identification of cancer-specific mutations and genes, prognostic and predictive biomarkers for therapy response	Enables personalized treatment plans; early detection and monitoring; comprehensive mutation analysis	High cost; large data volume requires advanced bioinformatics; interpretation of variants can be challenging	[77]
Cardiovascular Diseases	Genome-Wide Association Studies (GWAS), WGS, WES	Discovery of genetic variants associated with heart disease, biomarkers for risk assessments and therapeutic targets	Identification of high-risk individuals; potential for new therapeutic targets	Complex interplay of genetics and environment; data integration challenges	[78]
Neurodegenerative Diseases	WGS, WES, GWAS, Epigenetic Profiling	Identification of genetic mutations linked to Alzheimer’s, Parkinson’s, and other neurodegenerative diseases	Early diagnosis; understanding disease mechanisms; potential for targeted therapies	Genetic heterogeneity; need for large cohort studies	[79]
Diabetes	GWAS, WGS, WES, Transcriptomics	Genetic markers associated with insulin resistance, beta-cell function and complications of diabetes	Improved prediction of disease onset; potential for personalized treatment strategies	Complex genetic architecture; influence of lifestyle factors	[80]
Autoimmune Diseases	GWAS, WGS, Single Nucleotide Polymorphism (SNP) Analysis	Biomarkers for disease susceptibility, progression, and response to treatment in conditions like rheumatoid arthritis	Identification of susceptibility genes; tailored therapeutic interventions	Genetic and environmental interactions; variable disease phenotypes	[81]
Infectious Diseases	WGS, Pathogen Genomics, Metagenomics	Pathogen-specific genetic markers, host genetic factors influencing susceptibility and treatment response	Rapid pathogen identification; understanding pathogen-host interactions	High cost of sequencing; data complexity; need for rapid analysis turnaround	[82]
Rare Genetic Disorders	WGS, WES, Copy Number Variation (CNV) Analysis	Discovery of causative mutations and genes, development of diagnostic tests and personalized treatment approaches	Accurate diagnosis; potential for gene therapy; detailed understanding of rare diseases	High cost; difficulty in obtaining sufficient sample sizes; ethical considerations	[83]
Respiratory Diseases	WGS, Transcriptomics, Epigenomics	Identification of genetic variants linked to asthma, COPD and other respiratory condition biomarkers for disease management	Early detection; better disease management; identification of environmental and genetic interactions	Heterogeneous disease mechanisms; influence of environmental factors; data interpretation challenges	[84]

### 3.4. Proteomic Analysis in High-Throughput Pipelines

Proteomics is the large-scale study of proteins and their structures, functions and interactions within biological systems. This field is of crucial importance as proteins are the primary functional molecules in cells and influence almost all biological processes. Advances in mass spectrometry (MS) and other analytical techniques have significantly advanced the field, allowing the comprehensive identification and quantification of proteins from complex samples. These technologies allow researchers to study post-translational modifications, protein–protein interactions and the dynamic changes in protein expression under different conditions. Proteomics complements genomics by providing a more direct understanding of the functional state of a cell or organism and bridging the gap between genetic information and phenotypic expression. This integrative approach has profound implications for the understanding of disease mechanisms, the discovery of biomarkers and the development of targeted therapies [85,86]. High-throughput proteomics relies on a range of advanced analytical techniques and technologies to study proteins and their functions in biological systems. Mass spectrometry (MS) is the cornerstone of proteomics and enables the identification and quantification of proteins in complex biological samples. Techniques such as electrospray ionization (ESI) and matrix-assisted laser desorption/ionization (MALDI) are often used in conjunction with MS to ionize proteins and peptides for analysis. These methods enable the detection of low-abundance proteins and the identification of post-translational modifications that are critical for understanding protein function and regulation. In addition, high performance liquid chromatography (HPLC) is often used for protein separation prior to MS analysis, improving the resolution and accuracy of protein identification [85,87]. Recent advances in proteomics have also introduced methods such as top-down and bottom-up proteomics, which differ in their approach to protein analysis. Bottom-up proteomics, which involves digesting proteins into peptides prior to analysis, has gained acceptance due to its efficiency in processing complex mixtures. In contrast, top-down proteomics analyzes intact proteins, providing detailed information about protein isoforms and modifications. Techniques such as two-dimensional gel electrophoresis (2-DE) and tandem mass spectrometry (MS/MS) are used in these approaches to separate and identify proteins. In addition, quantitative proteomics techniques, including stable isotope labeling and label-free quantification, enable the precise measurement of protein abundance and dynamics under different biological conditions. Together, these methods enhance our ability to perform comprehensive proteomic analysis and facilitate biomarker discovery and the elucidation of disease mechanisms [85]. Techniques such as multiple reaction monitoring (MRM) and tandem mass spectrometry (MS/MS) enable high-throughput analysis of potential biomarkers and facilitates the identification and validation of disease-specific molecular signatures. For example, MS has been used extensively in cancer research to identify novel protein biomarkers that can distinguish between different cancer types and stages, providing valuable insights for early diagnosis and targeted treatment [88,89]. Data-independent acquisition (DIA) has become an important technique in proteomics and peptidomics as it enables comprehensive and reproducible quantification of proteins and peptides in complex biological samples. One of the most important tools for processing DIA data is the DIAproteomics pipeline, which integrates established software, such as OpenSwathWorkflow (http://openswath.org/, accessed on 1 June 2024) and PyProphet (https://github.com/PyProphet/pyprophet, accessed on 1 June 2024), to accomplish tasks ranging from spectral library searches to false discovery rate assessments. This pipeline supports the generation of spectral libraries from existing data-dependent acquisition (DDA) data and includes functions for retention time matching and chromatogram matching. The output consists of annotated tables and diagnostic visualizations that facilitate the interpretation of data and the calculation of fold-changes under different experimental conditions. Such robust data processing functions are essential for obtaining meaningful biological insights from large DIA datasets [90].

Another notable advance in DIA data processing is the development of DIA-NN, a software suite that utilizes deep neural networks to improve the identification and quantification performance of DIA experiments. DIA-NN implements advanced quantification and signal correction strategies that significantly improve the depth and accuracy of proteome coverage. This tool is particularly beneficial for high-throughput applications as it enables fast and safe proteomic analysis even with fast chromatographic methods. In addition, DIA-NN’s deep learning algorithms facilitate the deconvolution of complex DIA spectra and enable the identification of low-abundance proteins that might otherwise be overlooked. This capability is crucial for applications such as cancer biomarker discovery, where the detection of subtle changes in protein expression can lead to important clinical insights [91].

The application of MS in biomarker discovery is not limited to proteomics, but also extends to metabolomics and lipidomics, where it helps in the identification of biomarkers from small molecules associated with various diseases. High-resolution MS techniques, such as Fourier transform ion cyclotron resonance (FT-ICR) and Orbitrap, provide exceptional mass accuracy and resolution, enabling the precise characterization of metabolites and lipids. This capability is particularly valuable for understanding metabolic disorders and identifying metabolic pathways that are altered in disease, enabling the profiling and imaging of tissue samples and providing information on the spatial distribution of biomolecules directly from tissue sections, which is invaluable for disease diagnosis and biomarker discovery [92]. In addition, advances in MS technologies, such as imaging mass spectrometry (IMS), have enabled researchers to visualize the localization of drugs and their metabolites in tissues, providing insights into drug distribution and mechanism of action. This technique is particularly useful in pharmacology and toxicology, where understanding the interaction between drugs and biological tissues is crucial [93].

In peptidomics, MS plays a crucial role in the identification and characterization of bioactive peptides, i.e., short sequences of amino acids with significant biological activities. Peptidomics focuses on peptides derived from the degradation or processing of larger proteins, often revealing biomarkers and therapeutic targets. Advanced MS techniques combined with bioinformatics tools enable the detection and quantification of these peptides with high sensitivity and specificity. For example, MS-based peptidomics has contributed to the discovery of new biomarkers for neurodegenerative diseases such as Alzheimer’s disease by analyzing peptide patterns and post-translational modifications in patient samples [94,95]. The ability to perform detailed peptide mapping and quantification improves our understanding of disease pathology and supports the development of targeted interventions.

Proteomics and peptidomics analysis in high-throughput pipelines involves the systematic study of proteins, their structures, functions and interactions on a large scale, facilitated by advanced technologies and automated workflows. These pipelines utilize mass spectrometry (MS) as a core technique that enables the identification and quantification of thousands of proteins from complex biological samples. The development of sophisticated software platforms, such as the High-Throughput Autonomous Proteomic Pipeline (HTAPP), has markedly enhanced the efficiency and precision of proteomic studies. These platforms automate the entire process, from data acquisition to post-acquisition tasks, including peptide quantification, database searching and statistical validation. This enables researchers to process large datasets with minimal manual effort [96,97]. Automated pipelines, such as FAIMS-DIA (asymmetric high-field waveform ion mobility spectrometry—data-independent acquisition), have shown remarkable improvements in proteomic depth and reproducibility, identifying over 8000 proteins per MS acquisition. These advances enable the detailed profiling of cellular proteomes, even in studies of induced pluripotent stem cell-derived neurons. Such high-throughput proteomics pipelines are of central importance not only for basic biological research, but also for clinical applications such as biomarker discovery and drug development. They facilitate the analysis of changes in protein expression in response to disease states or therapeutic interventions, providing crucial insights into disease mechanisms and potential treatment strategies [98].

The integration of proteomics and peptidomics with other omics data is essential for a comprehensive understanding of biological systems and disease mechanisms. By combining proteomics with genomics, transcriptomics, metabolomics and epigenomics, researchers can construct detailed molecular networks that provide insights into cellular processes and interactions. For example, the integration of proteomic and genomic data can reveal how genetic variations influence protein expression and function, which is crucial for understanding disease progression and identifying therapeutic targets (Table 6). This multi-omics approach enables the identification of biomarkers and molecular signatures that are more robust and accurate than those obtained from single-omics analyses. Tools such as Omics Integrator and mixOmics facilitate the integration and interpretation of complex datasets, enabling researchers to gain new biological insights and develop more effective treatments [99,100].

The integration of proteomics data with other omics data also presents some challenges. These include dealing with heterogeneous data types and the need for advanced computational methods. Different omics technologies generate data in different formats, scales and dimensions, which complicates the integration process. Techniques such as Two-way Orthogonal Partial Least Squares (O2PLS) and machine learning algorithms are used to manage this complexity and provide robust frameworks for data integration and analysis. The LinkedOmics database, for example, integrates proteomic data with genomic and transcriptomic data from cancer studies, providing a comprehensive resource for exploring the molecular basis of cancer and identifying potential biomarkers and therapeutic targets. Such integrative approaches are crucial to the advancement of personalized medicine as they enable the development of targeted treatments based on a holistic understanding of the molecular mechanisms underlying disease [101,102].

A notable example of a multifunctional, automated high-throughput pipeline is the HTAPP (High-Throughput Autonomous Proteomic Pipeline), which was developed for proteomic analysis. HTAPP automates the entire process from peptide quantification to spectral database searching and statistical validation, significantly reducing the need for manual intervention and improving the reproducibility of results. This pipeline has been successfully used in various studies and has proven its robustness and efficiency in processing large proteomic data. For example, HTAPP has facilitated the identification and quantification of proteins in complex biological samples, leading to the discovery of new biomarkers and therapeutic targets in cancer research [96].

The Chromosome-Centric Human Proteome Project (C-HPP) [103] aims to explore the diversity of the human proteome, including rare variants, with a focus on liver tissue, HepG2 cells and plasma. Utilizing the proteogenomic approach, this powerful method predicts and validates proteoforms resulting from alternative splicing, mutations and transcript editing. A Python-based pipeline, PPLine, was developed to automate the discovery of single amino acid polymorphisms (SAPs), indels and alternatively spliced variants from raw transcriptome and exome sequencing data to predict proteotypic peptides. In this study [103], deep transcriptome sequencing of HepG2 cells and liver tissue, using Illumina HiSeq and Applied Biosystems SOLiD platforms, detected 7756 SAPs and indels, including 659 new variants. The study also found 17 indels in transcripts associated with alternative reading frames (ARFs) and identified novel proteoforms encoded by 203 liver and 475 HepG2 genes. These results form the basis for further proteomic studies by the C-HPP consortium [103].

Another important case study is the use of V-pipe to assess the genetic diversity of viruses. V-pipe is a bioinformatics pipeline specifically designed to process high-throughput sequencing data from viral genomes [104]. It combines various statistical models and computational tools to automate the analysis of raw sequencing data, including quality control, read mapping, alignment and low-frequency mutation detection. V-pipe has been instrumental in understanding the genetic diversity of virus populations within the host, which is critical for the study of virus transmission, virulence and pathogenesis. The ability of this pipeline to provide high quality read alignments and support benchmarking capabilities ensures accurate and reproducible results, making it a valuable tool in both research and clinical settings [104].

## 4. Integration and Interoperability of Omics Data

The integration and interoperability of omics data are critical to the advancement of biological research and enable a holistic understanding of complex biological systems. Effective integration combines different omics datasets, such as genomics, transcriptomics, proteomics and metabolomics, to provide comprehensive insights that individual data types alone cannot achieve. Strategies for data integration include early, mixed, intermediate, late and hierarchical integration, each of which offers unique advantages depending on the research context [59]. Successful integration methods must overcome challenges such as heterogeneity of data, management of large datasets and standardization of biological identities [105,106]. In addition, novel approaches, such as regularized canonical correlation analysis and graphical lasso, have shown promise in identifying important biomarkers and deciphering complex biological interactions [107]. As omics technologies continue to evolve, robust integration methods are essential to maximize the utility of multi-omics datasets and advance precision medicine and systems biology (Table 7) [12].

Effective data integration strategies are critical for promoting the interoperability of omics data and enabling comprehensive insights into complex biological systems. One prominent approach is early integration, in which all omics datasets are merged into a single matrix for subsequent analysis using machine learning models. This method utilizes the full spectrum of the data, but can be computationally intensive due to the high dimensionality [59]. Another approach is mixed integration, where each omics dataset is independently transformed into a new representation before being combined for downstream analysis, striking a balance between computational efficiency and data dimensionality [59].

Intermediate and late integration strategies provide additional flexibility by converting datasets into general and omics-specific representations simultaneously, or by analyzing each omics dataset separately before combining their predictions. Hierarchical integration, where datasets are organized based on previous regulatory relationships, provides a structured approach to data combination and improves the interpretability and relevance of results [59]. Omics data integration using regularized canonical correlation analysis and graphical lasso has shown promise in identifying important biomarker candidates and deciphering complex interactions within biological systems [107]. As high-throughput technologies continue to evolve, these integration strategies are crucial to maximizing the utility of multi-omics datasets and advancing precision medicine and systems biology.

Omics data integration and interoperability requires advanced tools and techniques to manage the complexity and heterogeneity of high-throughput datasets. One of the most important tools is the Omics Integrator software (https://fraenkel-nsf.csbi.mit.edu/omicsintegrator/, accessed on 1 June 2024), which uses network-based approaches to integrate different omics data and identify the underlying molecular pathways. This software uses advanced network optimization algorithms to find subnetworks with high confidence, facilitating the interpretation of multi-omics data in a biologically meaningful way [10]. Another notable tool is the OmicsPLS package, which implements Two-way Orthogonal Partial Least Squares (O2PLS) for efficient handling and integration of low and high dimensional datasets. This package enables inspection and visualization of integrated omics data, improving the ability to gain meaningful biological insights [108].

**Table 7:** Integration and Interoperability of Omics Data: Approaches, Tools, and Benefits.

Omics Type	Integration Approach	Interoperability Tools and Standards	Benefits	Limitations
Genomics	Cross-referencing genetic variants	VCF (Variant Call Format), dbSNP [109], Ensembl [110]	Identifies genetic variations and their effects	High data volume; interpretation of variants; privacy issues
Transcriptomics	Aligning RNA-seq data with genome	FASTQ, SAM/BAM, GTF/GFF, GEO (https://genome.ucsc.edu/ENCODE/, accessed on 1 June 2024)	Reveals gene expression patterns and alternative splicing	Data complexity; variability between samples
Proteomics	Correlating protein levels with gene expression	mzML [111], PRIDE [57], UniProt [112]	Understands protein abundance and functional roles	Sensitivity to sample preparation; high technical variability
Metabolomics	Linking metabolites to metabolic pathways	mzTab, HMDB [113], KEGG [114]	Provides insights into cellular metabolism and pathway activities	Complex data integration; diverse chemical properties
Epigenomics	Mapping epigenetic modifications	BED, WIG, GEO, ENCODE (https://genome.ucsc.edu/ENCODE/, accessed on 1 June 2024)	Studies DNA methylation, histone modifications and chromatin accessibility	High data complexity; dynamic nature of epigenetic changes
Lipidomics	Profiling lipid species	LIPID MAPS [115], mzXML [111]	Explores lipid composition and its role in cell biology	Heterogeneity of lipids; difficulty in quantifying low-abundance lipids
Glycomics	Characterizing glycan structures	GlyTouCan [116], UniCarb-DB [117]	Analyzes glycan functions and interactions	Structural complexity of glycans; limited analytical standards
Microbiomics	Integrating microbiome with host data	QIIME [118], MG-RAST [119]	Examines microbial communities and their impact on host health	Variability in sample preparation; complex data interpretation
Phenomics	Associating phenotypic traits with molecular data	PhenX [120], PheWAS [121], dbGaP [122]	Identifies molecular markers linked to phenotypes	High variability in phenotype data; integration with genomic data
Pharmacogenomics	Linking drug response to genetic profiles	PharmGKB [123], CPIC	Personalizes medicine based on genetic profiles	Ethical concerns; variability in drug response among individuals

In addition to these tools, various techniques have been developed to improve the integration of multi-omics data. XML is often used to facilitate cross-platform data integration as it is easy to learn, store and transfer. Techniques such as bio-warehousing, database federation and controlled vocabularies also play a crucial role in managing heterogeneous data sources [124]. In addition, integration strategies such as early, mixed, medium, late and hierarchical integration meet different research needs and increase the flexibility and applicability of data integration processes [59]. As this field progresses, the development of more robust, scalable and user-friendly tools and techniques will be crucial to fully exploit the potential of multi-omics data in biological research and precision medicine.

The integration and interoperability of omics data is a major challenge due to the heterogeneity and complexity of the datasets involved. One of the biggest challenges is the heterogeneity of the data, which includes different formats, scales and types of omics data such as genomics, proteomics and metabolomics. This diversity makes it difficult to bring these datasets together in a coherent framework for analysis. Furthermore, the amount of data generated by high-throughput technologies exacerbates these problems and makes data storage, management and retrieval complex tasks [125]. Another major challenge is the lack of standardization of different data types and sources, which hinders the effective sharing and reuse of data. Standardization issues include differences in terminology, units of measurement and data formats, which can lead to difficulties in integrating and interpreting data [12].

To overcome these challenges, various solutions have been proposed and developed. One effective approach is to use advanced bioinformatics tools and algorithms that can handle the complexity and scale of multi-omics data. For example, tools such as Omics Integrator and OmicsPLS facilitate the integration of different omics datasets by using sophisticated algorithms to identify meaningful biological pathways and interactions [10]. In addition, the use of standardized data formats and controlled vocabularies can significantly improve data interoperability. For example, XML-based data integration techniques provide a way to standardize the storage, migration and validation of omics data so that it can be more easily integrated and analyzed across different platforms. In addition, the use of machine learning and deep learning techniques has shown promise for managing and interpreting large-scale multi-omics data. They offer new insights into complex biological systems and improve the possibilities of predictive modeling [126].

An exemplary initiative in this area is NeDRexDB [127], which integrates data from a variety of biomedical databases such as OMIM [128], DisGeNET [129], UniProt [112], NCBI gene info, IID [130], Reactome [131], MONDO [132], DrugBank [133] and DrugCentral [134]. By aggregating data from these different sources, NeDRexDB constructs heterogeneous networks that represent different biomedical entities (e.g., diseases, genes, drugs) and their interconnections [127]. Researchers can access and explore these networks via NeDRexApp, NeDRexAPI [127] and the Neo4j endpoint [135] to NeDRexDB, creating a versatile platform for data analysis [127]. NeDRexApp, a Cytoscape [136] application, extends this functionality by providing implementations of state-of-the-art network algorithms, such as Multi-Steiner Trees (MuST) [137], TrustRank [138], Biclustering Constrained by Networks (BiCoN) [139] and Disease Module Detection (DIAMOnD) [140]. These advanced algorithms are accessible to users via the RESTful API and the user-friendly NeDRexApp interface. They require a list of user-selected genes (called seeds) as a starting point, with BiCoN being an exception as it uses gene expression data. These seeds can contain all or a subset of genes associated with a particular disease (disease genes) or genes within disease modules. In addition, expert knowledge can be used to select the seeds and the results can be statistically validated by calculating empirical *p*-values. In this way, NeDRex enables state-of-the-art network medicine methods that allow researchers in pharmacology and biomedicine to use their expertise to discover candidates for drug repurposing and gain deeper insights into disease mechanisms. By integrating diverse datasets and applying sophisticated computational tools, NeDRex exemplifies how advanced bioinformatics solutions can overcome the challenges posed by the heterogeneity and complexity of multi-omics data and ultimately advance precision medicine and biomedical research. A recent study has illustrated the capabilities of NeDRex by identifying critical modules involved in glioblastoma multiforme (GBM) disease, such as MYC, EGFR, PIK3CA, SUZ12 and SPRK2. In addition, hub genes were identified in a comprehensive interaction network containing 7560 proteins associated with GBM disease and 3860 proteins associated with signaling pathways involved in GBM. By integrating these results and performing a centrality analysis, 11 key genes involved in GBM disease were identified [141].

The integration of high-throughput omics data into precision medicine has significant implications for matching treatments to individual patients. By combining different types of data—such as genomics, proteomics, metabolomics and transcriptomics—researchers can create comprehensive molecular profiles that improve the understanding of disease mechanisms. This multi-omics approach enables the identification of specific biomarkers and therapeutic targets and facilitates personalized treatment plans that are more effective and have fewer side effects. In oncology, for example, the integration of multi-omics data has led to the discovery of novel cancer subtypes and the development of targeted therapies that improve patient outcomes [142]. In addition, advanced computational techniques, such as machine learning and deep learning, are being used to analyze these complex datasets and further improve the precision of therapeutic interventions [143]. Furthermore, the application of multi-omics data integration in precision medicine extends beyond cancer to other complex diseases. This holistic approach enables the stratification of patients based on their molecular profiles, leading to more precise diagnoses and tailored treatments. In the field of metabolic diseases, for example, the integration of multi-omics data has enabled the identification of key metabolic pathways and regulatory networks that can be targeted therapeutically [80]. In addition, the integration of electronic health records (EHR) with multi-omics data supported by artificial intelligence has the potential to revolutionize patient care by providing real-time, data-driven insights into disease progression and treatment efficacy [144]. These advances underscore the transformative impact of integrating high-throughput omics on the practice of precision medicine, which will ultimately lead to more personalized and effective healthcare solutions.

## 5. Future Directions

Future directions in the application and impact of high-throughput pipeline-omics integration will further revolutionize precision medicine through various innovative approaches and technological advances. A major focus will be on the development of more sophisticated computational tools and algorithms capable of integrating large and diverse datasets from genomics, transcriptomics, proteomics and metabolomics. The use of advanced machine learning and artificial intelligence techniques will be crucial to manage the complexity and heterogeneity of these datasets and enable more accurate predictions and insights into disease mechanisms and treatment responses [106]. In addition, further development of user-friendly platforms, such as MOMIC and OmicsSuite, will facilitate the broader application of multi-omics approaches in clinical and research settings by providing comprehensive and accessible tools for data analysis and visualization [145,146].

Another promising direction is the integration of omics data with electronic health records (EHR) and other real-world data sources, which can provide a more holistic view of patients’ health statuses and disease progressions. This approach can significantly improve personalized medicine by allowing continuous monitoring of patient health statuses and the dynamic adjustment of treatment plans based on real-time data. In addition, advances in high-performance computing and big data analytics will be crucial for handling the huge datasets generated by multi-omics studies, enabling faster and more efficient processing and interpretation of data [147]. Furthermore, the integration of spatial and single-cell omics technologies is expected to provide deeper insights into cellular heterogeneity and the spatial organization of tissues, opening new avenues for understanding complex biological processes and disease mechanisms [80]. Together, these advances will push the boundaries of precision medicine, enabling more accurate and personalized treatment strategies that improve patient outcomes and the overall efficiency of healthcare delivery.

## 6. Conclusions

High-throughput omics technologies have fundamentally changed biological research and offer unprecedented insights into the complexity of living systems. This review details the different types of omics—genomics, transcriptomics, proteomics, metabolomics and epigenomics—each of which contributes unique perspectives and valuable data to our understanding of biology. Advances in next-generation sequencing, mass spectrometry and microarray technologies have greatly improved the accuracy, efficiency and scope of data collection. The integration and analysis of this data, supported by robust bioinformatics tools and statistical methods, have become essential for managing the vast amounts of information generated by these technologies. The effective storage, sharing and analysis of data has opened up new avenues for research and collaboration, accelerating discoveries in disease research, drug development, agriculture and environmental studies. Despite remarkable progress, high-throughput omics still faces several challenges, including technical limitations, data complexity and cost and ethical issues. Overcoming these challenges requires continued innovation, interdisciplinary collaboration and the development of standardized protocols and ethical guidelines. Looking to the future, the integration of multi-omics data promises holistic insights into biological systems, personalized medicine and a better understanding of complex diseases and environmental interactions. As technologies and methods evolve, high-throughput omics will remain at the forefront of scientific discovery and shape the future of biological and biomedical research.

## Figures and Tables

**Table 1:** Overview of High-Throughput Omics Technologies.

Omics Type	Approaches	Outputs	Goals	Practical Suggestions
Genomics	High-throughput sequencing, annotation tools	Genome sequences, genetic variants	Identify genetic mutations, understand disease genetics	Use integrated databases (e.g., Ensembl) for annotation
Transcriptomics	RNA sequencing, normalization, analysis tools	Gene expression profiles, splicing variants	Analyze gene expression changes, understand regulatory mechanisms	Combine with single-cell RNA-seq for detailed insights
Proteomics	Mass spectrometry, protein databases	Protein identification, quantification	Understand protein functions, identify biomarkers and targets	Employ bioinformatics tools (e.g., MaxQuant) for data processing
Metabolomics	NMR spectroscopy, mass spectrometry	Metabolite profiles, metabolic pathways	Identify metabolic changes, understand pathways and disease mechanisms	Use MetaboAnalyst for comprehensive analysis
Epigenomics	DNA methylation analysis, ChIP-Seq	Epigenetic modification maps	Study gene regulation, understand epigenetic influences on disease	Integrate data with ENCODE for broader insights
Lipidomics	Mass spectrometry, chromatography	Lipid profiles, lipid interactions	Investigate lipid metabolism, understand lipid-related diseases	Utilize LipidSearch for detailed lipid analysis
Glycomics	Mass spectrometry, lectin microarrays	Glycan structures, glycosylation patterns	Study glycosylation processes, understand glycan roles in health	Use GlycoWorkbench for structural analysis
Microbiomics	16S rRNA sequencing, metagenomics	Microbial community composition	Understand microbiome contributions to health, study microbial interactions	Integrate with QIIME for diversity analysis
Metagenomics	High-throughput sequencing, data integration tools	Community genomic data, functional profiles	Analyze microbial communities, understand environmental impacts	Use MG-RAST for metagenomic analysis
Pharmacogenomics	Genotyping, pharmacokinetic studies	Genetic markers for drug response	Personalize medicine, predict drug response and side effects	Apply PharmGKB resources for clinical interpretation
Toxinomics	Toxicogenomics, proteomics, metabolomics	Toxicity profiles, biomarker identification	Assess environmental and drug toxicities, identify toxicity mechanisms	Utilize ToxPi for visualizing risk assessments
Exposomics	Environmental monitoring, high-throughput sequencing	Exposure biomarkers, environmental impacts	Understand effects of environmental exposures on health	Integrate exposomic data with health records
Single-cell Omics	Single-cell sequencing, advanced imaging	Single-cell gene expression, protein profiles	Investigate cellular heterogeneity, understand cell functions	Use Seurat for single-cell data analysis
Spatial Omics	Spatial transcriptomics, proteomics imaging	Spatial maps of gene/protein expression	Analyze tissue architecture, understand spatial organization	Apply 10× Genomics Visium for spatial transcriptomics
Nutrigenomics	Diet records, genotyping	Gene-diet interactions, nutritional biomarkers	Understand diet impact on gene expression, personalize nutrition	Combine with dietary intake software for comprehensive plans
Immunomics	Immune profiling, sequencing	Immune cell profiles, cytokine levels	Study immune responses, understand autoimmune diseases	Utilize Cytobank for immune profiling analysis

**Table 3:** Challenges and Opportunities in High-Throughput Omics Technologies.

Aspect	Challenges	Opportunities	Requirements	Pitfalls
Data Complexity	Managing large volumes of heterogeneous data	Development of advanced bioinformatics tools for data integration	Sophisticated bioinformatics tools and infrastructures	Data heterogeneity and lack of standardized data formats
Technical Limitations	Sensitivity, specificity and accuracy of high-throughput technologies	Continuous technological advancements improving precision	Advanced instrumentation and accurate measurement techniques	Overfitting and varying samples/instrument quality
Cost and Accessibility	High costs of equipment and reagents	Reduction in costs through technological innovations and economies of scale	Cost-effective technological solutions	High initial investment and maintenance costs
Data Storage and Management	Efficient storage, retrieval and sharing of large datasets	Cloud-based storage solutions and standardized data formats	Robust data storage and management systems	Data losses and breaches in data security
Data Interpretation	Complexity in analyzing and interpreting multi-omics data	Use of machine learning and AI for comprehensive data analysis	Expertise in machine learning and data analytics	Misinterpretations due to data complexity
Ethical and Privacy Concerns	Ensuring privacy and security of sensitive genetic information	Development of ethical guidelines and robust security measures	Adherence to ethical standards and data protection protocols	Privacy breaches and ethical dilemmas
Interdisciplinary Collaboration	Need for expertise in multiple disciplines (biology, chemistry and bioinformatics)	Fostering collaborations across diverse fields to drive innovation	Strong collaborative frameworks and interdisciplinary training	Miscommunications and integration issues among diverse teams
Standardization	Lack of standardized protocols and methodologies	Establishing universal standards for reproducibility and comparability	Universal standards and protocols	Inconsistent results and lack of comparability across studies

**Table 4:** Examples of text mining tools are.

Tool	Description	Key Applications
MetaMap [35]	Maps biomedical text to the Unified Medical Language System (UMLS) Metathesaurus, identifies concepts in biomedical literature	Data annotation and information retrieval
Textpresso [36]	Information retrieval and extraction system for biological literature, categorizes and indexes text based on biological concepts	Facilitates extraction of specific information from biological literature
Pubpular [37]	Identifies high-priority proteins in human diseases based on semantic similarity, analyzes text data from biomedical literature	Prioritizes proteins relevant to disease pathology by calculating semantic similarities between protein and disease terms
VoSviewer [38]	Software tool for creating and visualizing bibliometric networks, analyzes co-occurrence of terms in scientific literature	Identifies research trends, collaborations and key areas of interest in the biomedical field
PubTator [34]	Provides annotations for biomedical entities such as genes, diseases and chemicals in text	Extracts relevant information from large corpora of biomedical literature

**Table 6:** Applications of Biomedical Mass Spectrometry in Proteomics and Peptidomics.

Application Area	Proteomics	Peptidomics	Advantages	Limitations
Protein Identification	Identifying proteins in complex mixtures	Identifying peptide sequences and modifications	Comprehensive identification of proteins and their functions	Complex sample preparation and high data complexity
Quantitative Analysis	Measuring protein expression levels	Quantifying peptide abundances	Accurate quantification of protein expression levels	Requires high sensitivity and precision in measurement
Post-Translational Modifications	Detecting phosphorylation, glycosylation and acetylation	Characterizing modifications on peptides	Detailed analysis of protein modifications	Complex detection and interpretation of multiple modifications
Biomarker Discovery	Identifying protein biomarkers for diseases	Discovering peptide biomarkers for diagnostic purposes	Potential to discover novel biomarkers for various diseases	Validation of biomarkers is resource-intensive and time-consuming
Protein-Protein Interactions	Analyzing interaction networks	Studying peptide-mediated interactions	Insight into protein interaction networks and cellular processes	Difficult to detect transient or weak interactions
Structural Proteomics	Investigating protein structures and conformations	Analyzing peptide structures and dynamics	Understanding protein folding, stability and interactions	Requires advanced techniques like X-ray crystallography or NMR
Clinical Proteomics	Profiling proteins in clinical samples (e.g., blood, tissue)	Profiling peptides in clinical samples	Direct application to clinical diagnostics and personalized medicine	Variability in clinical samples can affect reproducibility
Proteome Mapping	Mapping the entire proteome of organisms or cells	Mapping peptidomes for specific conditions	Comprehensive overview of proteome composition and changes	Requires extensive data analysis and integration
Drug Development	Identifying drug targets and mechanisms	Screening peptide-based therapeutics	Identification of novel drug targets and understanding mechanisms of action	High cost and time investment in drug discovery pipeline
Pathway Analysis	Studying protein roles in biological pathways	Analyzing peptides involved in signaling pathways	Insight into biological pathways and their regulation	Complexity of pathway interactions and need for high-throughput analysis
Immune Monitoring	Profiling immune-related proteins	Characterizing antigenic peptides	Identification of immune responses and potential vaccine targets	High variability and need for extensive validation
De Novo Sequencing	Sequencing unknown proteins	Sequencing unknown peptides	Determining the sequence of novel proteins and peptides	Requires advanced algorithms and high-quality mass spectrometry data

## Data Availability

Not applicable.
